# Demographic and economic disparities in hand surgical procedures in Brazil: Analysis of DATASUS data

**DOI:** 10.31744/einstein_journal/2026GS2322

**Published:** 2026-09-09

**Authors:** Lucas Ferracini Viotto, Tiago Lorenzi da Rocha e Silva, Mariana Cristina Apude, Tiago Guedes da Motta Mattar, Vinícius Ynoe de Moraes, Raquel Bernardelli Iamaguchi, Gustavo Bersani Silva, Rames Mattar, Mario Vieira Guarnieri

**Affiliations:** 1 Hospital Israelita Albert Einstein São Paulo SP Brazil Hospital Israelita Albert Einstein, São Paulo, SP, Brazil.; 2 Universidade de São Paulo Faculdade de Medicina Hospital das Clínicas São Paul SP Brazil Hospital das Clínicas, Faculdade de Medicina, Universidade de São Paulo, São Paulo, SP, Brazil.

**Keywords:** Hand, Big Data, Epidemiology, Surgical procedures, operative, Health care costs

## Abstract

**Objective:**

To analyze epidemiological, demographic, and health cost data of major hand surgery procedures in Brazil's public health system and explore inequalities, regional utilization patterns, and economic impact to inform policy improvements.

**Methods:**

This cross-sectional study analyzed 1,026,582 hand surgery procedures from DATASUS (2018-2024). The data included distal radius fractures, amputations, replantations, carpal tunnel syndrome, and brachial plexus injuries linked to ICD-10 codes. The variables included sex, race/color, age, geographic region, and hospital costs. Statistical analysis employed one-way ANOVA, Tukey's test, χ^2^, Pearson's correlation, confidence intervals, and significance was set at p<0.05. The analysis used SPSS v.26, Minitab v.21.2, and Excel 2010.

**Results:**

Health procedures were predominantly performed in male patients (53.5%, n=549.641) and were concentrated in the Southeast region (38.1%, n=391,340). Significant regional variation was observed in total procedure costs, with higher mean values in the South (mean=458,82, SD=455,20, p<0.001), Southeast (mean=453.75, SD=458.93, p<0,001) and West-Central (mean=480,09, SD=446.24, p<0,001) regions. Urgent admissions accounted for 72% of cases, reflecting the traumatic profile of hand surgery within the Brazilian Public Health System. Regarding procedure type, regional disparities were evident, with high-complexity surgeries such as those for brachial plexus injuries costing most in the South (Mean=1295,61, SD=502.28, p<0.001), and low-complexity procedures (for carpal tunnel syndrome) showing higher mean costs in the South (Mean=476.55, SD=348.50, p<0.001) and West-Central regions (Mean=396.38, SD=160.04, p<0.001).

**Conclusion:**

DATASUS data showed that procedures were concentrated in the Southeast region (38.1%) and were predominantly performed in men (53.5%), with 72% of the procedures classified as urgent. Higher mean costs were noted in the West-Central/Southeast/South than in the North/Northeast. Brazil requires adequate infrastructure and training to ensure equitable access to healthcare.

## In Brief

An analysis of 1,026,582 hand surgeries in Brazil's SUS (2018-2024) revealed regional disparities. The findings showed male predominance (53.5%), a 72% urgency rate, and higher costs in the Central-West, South, and Southeast regions. The results highlight the need for equitable health policies and improved resource allocation nationwide.

## Highlights

An analysis of over one million surgeries revealed regional access disparities in Brazil.

A total of 72% of procedures were urgent, reflecting the traumatic profile in the SUS

The mean costs were higher in the West-Central, South, and Southeast regions (p<0.001).

The male predominance (53.5%) highlights the socioeconomic impact on workers.

## Graphical abstract

**Figure:**
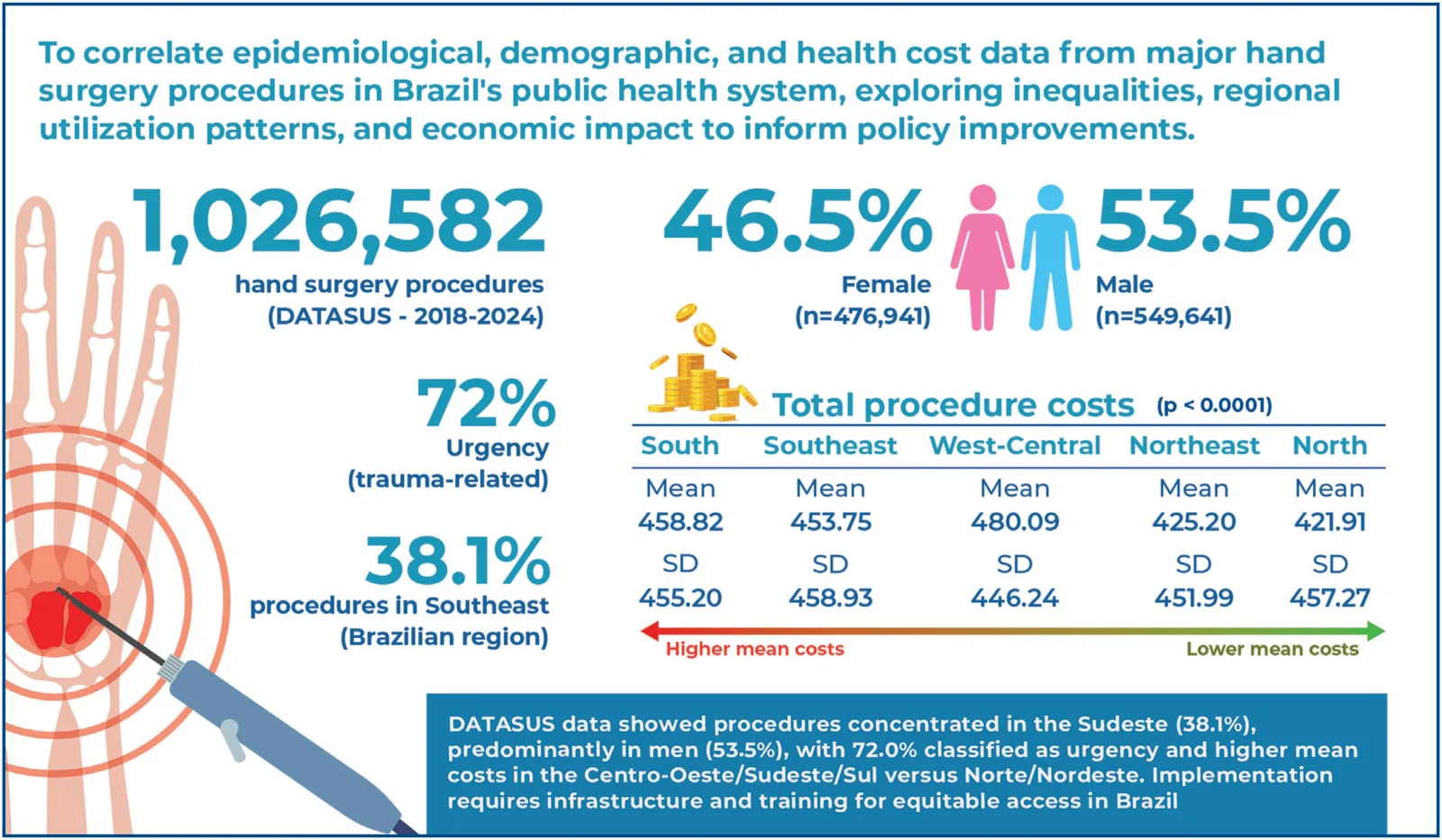

## INTRODUCTION

The Brazilian Public Health System (SUS - *Sistema Único de Saúde*) is one of the largest and most complex public health systems in the world, covering primary care to high-complexity services, urgent and emergency care, and epidemiological surveillance.^(1)^ In this context, the Informatics Department of SUS (DATASUS - *Departamento de Informática do Sistema Único de Saúde*) plays a fundamental role in data integration and in providing strategic information, enabling planning, evaluation of public policies, and health management.^(2)^

Despite the potential of these databases, some limitations still persist. The coding of procedures and diagnoses does not always reflect the actual complexity of surgical treatment, as observed in ankle fractures, in addition to the lag in the adjustments of paid values in relation to inflation, resulting in discrepancies between costs and remuneration.^(3)^

This scenario is particularly relevant in orthopedic surgery. Distal radius fractures are very common, with significant economic and social impacts, being responsible for approximately 634,000 annual cases in the United States and costs exceeding 535 million dollars. The advent of volar locking plates has increased the rate of surgical indications, with consequent increases in direct treatment costs.^(4)^ Although surgery has a higher initial cost compared to conservative treatment, studies indicate benefit in terms of early return to work activities, suggesting favorable cost-effectiveness in certain patient profiles.^(5)^

Another highly prevalent condition is carpal tunnel syndrome, which is responsible for more than 500,000 annual procedures in the United States. Although the endoscopic technique is not widely available in SUS, international studies demonstrate that, despite the higher initial cost, it has greater cost-effectiveness by reducing time away from work. Additionally, the use of the WALANT technique has been shown to be advantageous in economic terms when compared to conventional anesthesia.^(6,7)^

In the field of trauma, amputations and replantations represent not only clinical challenges but also a significant economic and social burden. It is estimated that approximately 31,000 accidents with circular saws involving the thumb occur per year in the United States, a number that may be even higher in Brazil. More than 90% of amputations occur in the fingers, predominantly affecting young and productive workers, with economic repercussions for the individual and the public health system.^(8,9)^

Given this scenario, it is necessary to understand the epidemiological data, costs, and geographic distribution of the main hand surgery procedures in Brazil. The use of DATASUS allows the investigation of these variables on a national scale, identifying regional inequalities, patterns of utilization, and economic implications.

## OBJECTIVE

To analyze epidemiological information, geographic distribution, and costs of major surgical procedures in hand surgery in Brazil using a national database (DataSUS) in order to identify regional inequalities, utilization patterns, and economic impact, and to generate evidence that supports the improvement of public policies.

## METHODS

As this study was based on anonymous and widely available data, it was exempt from evaluation by a research ethics committee (CNS Resolution 510/2016).

This retrospective study included 1,026,582 data points and high variability. It employed data extracted from DATASUS between 2018 and 2025, which covers the entire country and is publicly available on the Tabnet platform. Information on the demographic and epidemiological distribution of distal radius fractures, amputations and replantations, carpal tunnel syndrome, brachial plexus injuries, and other hand surgical procedures through ICD-10 codes (Table 1S, Supplementary Material) is included. The variables analyzed included sex, race/color, age group, geographic region, and hospital costs.

After data collection, enumerated data were decoded into nominal data, and data such as patient municipality, establishment municipality, duration of hospitalization, reasons for discharge, surgical complexity, number of deaths, patient educational level, patient occupation, hospital management type, bed specialty, AIH number, and patient ZIP code were discarded from the analysis.

The extracted data were organized into spreadsheets and subsequently subjected to statistical analysis. Descriptive analyses included absolute and relative frequencies and measures of central tendency (mean and median) and dispersion (standard deviation and range). For comparison between groups, one-way ANOVA with Tukey's post-hoc test was used for continuous variables, and the chi-square test was used for categorical variables. Pearson's correlation was applied to evaluate associations between numerical variables. A significance level of p<0.05 was adopted. Confidence intervals (95%CI) were calculated for the means of the costs and other relevant parameters. The analyses were performed using SPSS version 26, Minitab version 21.2, and Microsoft Excel 2010, which are all recognized tools in health research, to ensure the robustness and reproducibility of the findings.

## RESULTS

### Demographic and epidemiological characteristics

This study analyzed 1,026,582 hand surgery procedures performed in the Brazilian SUS between 2018 and 2024. Demographic characteristics are summarized in tables 1 and 2.

**Table 1:** Sample characteristics of hand surgical procedures recorded in DATASUS (n=1,026,582)

Variable		n (%)
Region		
	West-Central	102,068 (9.9)
	Northeast	243,744 (23.7)
	North	63,654 (6.2)
	Southeast	391,340 (38.1)
	South	225,776 (22.0)
Race/Ethnicity		
	Yellow	13,973 (1.4)
	White	378,660 (36.9)
	Indigenous	1,425 (0.1)
	Mixed Race	375,851 (36.6)
	Black	31,837 (3.1)
	Not Reported	224,836 (21.9)
Sex		
	Female	476,941 (46.5)
	Male	549,641 (53.5)
Surgical Complexity		
	High	12,218 (1.2)
	Medium	1,014,364 (98.9)
Procedure Group		
	Upper Limb Amputations and Replantations	186,534 (18.2)
	Distal Radius Fracture	628,797 (61.3)
	Brachial Plexus Injury	9,052 (0.9)
	Carpal Tunnel Syndrome	195,466 (19.0)
	Hand Surgical Treatment	6,733 (0.7)
Type of Admission		
	Elective	287,104 (28.0)
	Urgent	739,478 (72.0)

Percentages are in relation to the total sample size.

Source: DATASUS (Ministry of Health, Brazil), 2018-2024.

**Table 2:** Quantitative characteristics of hand surgical procedures recorded in DATASUS (n=1,026,582)

Variable	Mean	Median	95%CI
Hospital costs (R$)	306.25	201.68	[305.48; 307.02]
Medical fees (R$)	129.83	105.92	[129.70; 129.96]
Total costs (R$)	448.81	347.62	[447.93; 449.69]
Age (years)	42.56	45	[42.52; 42.60]

Values expressed as cost per procedure in Brazilian reais (R$) or age in years, as indicated. 95% CI refers to the mean.

95% CI: 95% confidence interval.

Source: DATASUS (Ministry of Health, Brazil), 2018-2024.

A predominance of male patients was observed (53.5%; 95%CI=53.4-53.6%), indicating a sex-related imbalance in surgical demand. This pattern is consistent with the traumatic and occupational profiles of hand injuries and highlights a disproportionate burden among economically active male individuals.

The geographic distribution revealed a marked regional concentration. The Southeast region accounted for 38.1% of all procedures, whereas the North and West-Central regions combined represented only 16.1% of the national volume. These differences were statistically significant (p<0.001) and suggest unequal regional access to specialized hand surgical care within the SUS.

Racial self-classification showed similar proportions of white (36.9%) and mixed-race (36.6%) patients; however, a substantial proportion of the procedures (21.9%) lacked race/color information. This limitation restricts deeper inference but may itself reflect structural weaknesses in demographic data reporting within the public health system.

### Cost analysis: Brazil

Cost analysis demonstrated substantial heterogeneity, with coefficients of variation exceeding 50% for all cost components, indicating wide dispersion among procedure types and regions.

The national mean hospital cost was R$306.25 (95%CI=305.48-307.02), professional service cost was R$129.83 (95%CI=129.70-129.96), and total cost per procedure was R$448.81 (95%CI=447.93-449.69). Although these values represented national averages, they masked significant regional disparities (Table 3).

**Table 3:** Comparative analysis of total costs of hand surgical procedures stratified by Brazilian geographic region n=1,026,582)

Region	Mean (R$)	Standard deviation (R$)	n	p value
West-Central	480.09	446.24	102,068	<0.001
Northeast	425.20	451.99	243,744	<0.001
North	421.91	457.27	63,654	<0.001
Southeast	453.75	458.93	391,340	<0.001
South	458.82	455.20	225,776	<0.001

Values are expressed as the mean cost per procedure. p-values were obtained using one-way ANOVA.

R$: Brazilian reais.

Source: DATASUS (Ministry of Health, Brazil), 2018-2024.

### Regional and procedural cost disparities

Regional analysis of total costs revealed consistent disparities. The West-Central, Southeast, and South regions showed higher mean costs per procedure, whereas the North and Northeast regions exhibited systematically lower values (p<0.001). These findings suggest that the economic allocation for similar surgical procedures differs substantially across regions.

When stratified by procedure group, the disparities became more pronounced. High-complexity procedures, such as surgeries for brachial plexus injury, demonstrated the highest mean costs in the South, while other regions showed lower and more homogeneous values. Conversely, lower-complexity procedures, such as surgeries for carpal tunnel syndrome, exhibited marked regional variation, with the South and West-Central regions recording significantly higher mean costs than the North and Northeast regions (Table 4).

**Table 4:** Economic disparities in mean cost per hand surgical procedure by procedure group and geographic region in Brazil (n=1,026,582)

Procedure Group		Region	Mean (R$)	Standard Deviation	p value
Hand Surgical Treatment					
	West-Central	480.09	446.24	102.068	<0.001
	Northeast	425.20	451.99	243.744	<0.001
	North	421.91	457.27	63.654	<0.001
	Southeast	453.75	458.93	391.340	<0.001
	South	458.82	455.20	225.776	<0.001
Distal Radius Fracture					
	West-Central	507.61	429.26	67.065	<0.001
	Northeast	421.48	326.74	171.020	<0.001
	North	408.09	301.00	44.099	<0.001
	Southeast	483.68	421.58	219.006	<0.001
	South	441.97	368.64	127.607	<0.001
Upper Limb Amputations and Replantations					
	West-Central	433.15	576.49	18.543	<0.001
	Northeast	466.13	776.45	49.536	<0.001
	North	448.47	738.76	15.871	<0.001
	Southeast	420.19	728.20	68.366	<0.001
	South	446.04	764.33	34.218	<0.001
Brachial Plexus Injury					
	West-Central	1.246.30	468.18	592	<0.001
	Northeast	1.172.64	515.06	1.401	<0.001
	North	1.232.84	714.75	474	<0.001
	Southeast	1.188.35	564.41	4.548	<0.001
	South	1.295.61	502.28	2.037	<0.001
Carpal Tunnel Syndrome					
	West-Central	396.38	160.04	15.314	<0.001
	Northeast	363.06	100.68	20.703	<0.001
	North	375.79	141.59	2.791	<0.001
	Southeast	381.80	149.91	95.832	<0.001
	South	476.55	348.50	60.826	<0.001

Values are expressed as the mean cost per procedure in Brazilian reais (R$). p-values were obtained using one-way ANOVA

R$: Brazilian reais; 95% CI: 95% confidence interval.

Source: DATASUS (Ministry of Health, Brazil), 2018-2024.

These patterns indicate that regional inequities are not uniform across procedure types and disproportionately affect both high- and low-complexity hand surgical care.

### Admission type and trauma profile

Urgent admissions accounted for 72.0% of all procedures, reinforcing the predominantly traumatic profile of hand surgery procedures within the SUS. This high proportion contrasts with patterns observed in high-income countries, where elective procedures represent a larger share of hand surgical care, and suggests limited access to timely elective treatment in the Brazilian SUS.

## DISCUSSION

This analysis of more than one million procedures registered in DATASUS allowed the documentation of marked regional, racial, and sex disparities in hand surgical care provided by the SUS. The concentration of 38.1% of procedures in the Southeast region reflects demographic, historical, and structural inequalities in the Brazilian health system, as previously described in studies on other surgical specialties. ^(10,11)^

The predominance of males (53.5%) corroborates international literature, which attributes greater exposure to occupational trauma and risky activities to this sex.^(12,13)^ The concentration in the economically active age group (30-49 years) reinforces the socioeconomic impact of these injuries, potentially affecting the productive capacity and quality of life of these patients.

Regarding race/color, although 21.9% of records presented missing information, a known limitation of DATASUS,^(14)^ the similar distribution between White and Mixed-race patients contrasts with disparities observed in other areas of surgical care, where the Mixed-race and Black population historically had reduced access.^(15)^ However, these results should be interpreted with caution due to underreporting.

The finding that 72% of procedures were classified as urgent reinforces the predominantly traumatic nature of the conditions treated within the scope of hand surgery in the SUS. This profile differs from international patterns in developed countries, where elective procedures for degenerative conditions such as carpal tunnel syndrome represent a larger proportion.^(16,17)^

The significant regional variation in costs, with the Southeast region having substantially higher values than the North and Northeast regions, may reflect not only differences in cost of living, but also structural and technological disparities in healthcare provision. Studies on brachial plexus surgeries and peripheral nerve tumor treatments have already documented similar inequalities in the SUS.^(18,19)^

The high prevalence of distal radius fractures (28.5%) and carpal tunnel syndrome (22.3%) aligns with international epidemiology and reinforces the need for specific actions to prevent and treat these conditions.^(4,6)^ However, the lag in SUS remuneration values in relation to real costs, previously documented in other orthopedic procedures ^(20,21)^ may impact the availability and quality of services offered.

The present study has limitations inherent to the use of DATASUS, including possible coding errors, underreporting, and lack of clinical details about case complexity, type of rehabilitation, and functional outcomes.^(22)^

The incorporation of large databases into epidemiological analysis represents an opportunity to expand the understanding of regional, racial, and sex inequalities in hand surgery in Brazil. Future research should focus on the longitudinal evaluation of clinical outcomes (temporal analysis of data), impact of rehabilitation, new microsurgical techniques, and formulation of policies to minimize disparities and improve care for specific populations or clinical conditions.

## CONCLUSION

The national analysis based on DATASUS revealed marked demographic and economic disparities in hand surgical care in the Brazilian Public Health System, stratified by region, race, and sex. These findings reinforce the need for public policies to minimize inequalities, allocate resources to high-complexity procedures, and ensure training of specialized staff in Brazil.

## Data Availability

Data are available to reviewers upon request.
