# Supplementary material for: Demographic and economic disparities in hand surgical procedures in Brazil: Analysis of DATASUS data
Source: Einstein (Sao Paulo). 2026 Sep 9;24:eGS2322. doi: 10.31744/einstein_journal/2026GS2322 (PMC13682773; doi:10.31744/einstein_journal/2026GS2322)
Supplement: Supplementary file 1 [file 2317-6385-eins-24-eGS2322-Suppl01.pdf]

SUPPLEMENTARY MATERIAL

Demographic and economic disparities in hand surgical procedures in Brazil: Analysis of DATASUS data

Lucas Ferracini Viotto, Tiago Lorenzi da Rocha e Silva, Mariana Cristina Apude, Tiago Guedes da Motta Mattar, Vinícius Ynoe de Moraes, Raquel Bernardelli Iamaguchi, Gustavo Bersani Silva, Rames Mattar Junior, Mario Vieira Guarnieri

DOI: 10.31744/einstein_journal/2026GS2322

Table 1S. ICD-10 codes

**Distal radius fractures:**
S525	S526	S528
**Amputations and replantations:**
M212	S623	S624	S627	S650	S651	S653	S657	S670	S678	S680	S681	S682	S683	S684	S688	S689	S697	S908	S925	S927	S950	S951	S957	S958	S971	S978	S980	S981	S982	S983	S984	T233	T847	T871	T873	T874	T875	T876	T922	T924	T926
**Carpal tunnel syndrome:**
A300	A301	A302	A303	A304	A305	A308	A309	B92	G560	G561
**Surgical treatment of Hand:**
Q681	Q690	Q691	Q692	Q699	Q716	Q718	Q723	Q727	Q700	Q701	Q704	Q709
**Brachial Plexus Injury:**
G540	S143

Source: https://cremesp.org.br/
